# Conservation of pregnancy-specific glycoprotein (PSG) N domains following independent expansions of the gene families in rodents and primates

**DOI:** 10.1186/1471-2148-5-39

**Published:** 2005-06-29

**Authors:** Andrew S McLellan, Wolfgang Zimmermann, Tom Moore

**Affiliations:** 1 Department of Biochemistry, Biosciences Institute, University College Cork, College Road, Cork, Ireland; 2 Tumor Immunology Group, LIFE Center, University Clinic Grosshadern, Ludwig-Maximilians-University Muenchen, Marchioninistrasse 23, D-81377 Muenchen, Germany

## Abstract

**Background:**

Rodent and primate pregnancy-specific glycoprotein (*PSG*) gene families have expanded independently from a common ancestor and are expressed virtually exclusively in placental trophoblasts. However, within each species, it is unknown whether multiple paralogs have been selected for diversification of function, or for increased dosage of monofunctional PSG. We analysed the evolution of the mouse PSG sequences, and compared them to rat, human and baboon PSGs to attempt to understand the evolution of this complex gene family.

**Results:**

Phylogenetic tree analyses indicate that the primate N domains and the rodent N1 domains exhibit a higher degree of conservation than that observed in a comparison of the mouse N1 and N2 domains, or mouse N1 and N3 domains. Compared to human and baboon PSG N domain exons, mouse and rat PSG N domain exons have undergone less sequence homogenisation. The high non-synonymous substitution rates observed in the CFG face of the mouse N1 domain, within a context of overall conservation, suggests divergence of function of mouse PSGs. The rat PSG family appears to have undergone less expansion than the mouse, exhibits lower divergence rates and increased sequence homogenisation in the CFG face of the N1 domain. In contrast to most primate PSG N domains, rodent PSG N1 domains do not contain an RGD tri-peptide motif, but do contain RGD-like sequences, which are not conserved in rodent N2 and N3 domains.

**Conclusion:**

Relative conservation of primate N domains and rodent N1 domains suggests that, despite independent gene family expansions and structural diversification, mouse and human PSGs retain conserved functions. Human *PSG *gene family expansion and homogenisation suggests that evolution occurred in a concerted manner that maintains similar functions of PSGs, whilst increasing gene dosage of the family as a whole. In the mouse, gene family expansion, coupled with local diversification of the CFG face, suggests selection both for increased gene dosage and diversification of function. Partial conservation of RGD and RGD-like tri-peptides in primate and rodent N and N1 domains, respectively, supports a role for these motifs in PSG function.

## Background

In tandemly repeated gene families, in which all members share a common function, there is a tendency for concerted evolution that is characterised by homogenisation of gene sequences [1]. Classical examples include the histone and ribosomal RNA genes. In such cases the expansion of gene families is driven by selection for high expression [2]. Concerted evolution is generally maintained by unequal crossover, intergenic gene conversion or other illegitimate recombination mechanisms [1,2]. Conversely, there are multigene families whose members encode diverse functions e.g. genes encoding immunoglobulin (Ig), T cell receptor (TCR) and major histocompatibility complex (MHC) proteins [1]. Such diversity occurs when there is less homogenisation than mutation, due to the evolution of specific programmed mutational mechanisms [3]. In addition, more complex modes exist; for example, the immunoglobulin heavy-chain variable-region (V_H_) genes encode proteins with identical functions, but exhibit little concerted evolution [4]. Instead, their evolution is governed by divergence and a birth-and-death process of gene duplication and dysfunctioning mutations [2].

Similar to other families of highly expressed trophoblast-specific genes such as the pregnancy-associated glycoproteins (PAG) [5], the pregnancy-specific glycoproteins, which are the most abundant foetal proteins in the maternal bloodstream during human late pregnancy, are encoded by multiple tandemly arrayed genes [6,7]. The PSG family of glycoproteins, with the related CEA-related cell adhesion molecule (CEACAM) proteins, are part of the immunoglobulin superfamily [8]. The Ig domain structure of the human and mouse PSGs differs, as follows: Human PSGs contain one V-like Ig domain (N), C2-like Ig domains (A and B) and relatively hydrophilic tails (C), with domain arrangements classified as type I (N-A1-A2-B2-C), type IIa (N-A1-B2-C), type IIb (N-A2-B2-C), type III (N-B2-C) and type IV (A1-B2-C) [9]. In contrast, mouse PSGs typically have three or more N domains followed by a single A domain [7,10]. The common ancestor of rodent and primate PSGs and CEACAMs was probably similar to CEACAM1, which is the only CEA family member with an identical gene structure in the human, rat and mouse that encodes all types of extracellular domains present in CEACAM and PSG proteins. The time of initial gene duplication is estimated at 90 Myr [11], approximately the time of rodent-primate divergence. The independent expansion of human and mouse *PSG *gene families occurred through further gene duplication and exon shuffling events [7,12,13].

The independent expansion of *PSG *gene families in rodents and primates indicates convergent evolution, implying that PSG function is conserved. These events can be interpreted in the context of evolutionary theories of parent-offspring and inter-sibling conflicts that promote transcriptional 'arms races' leading to high expression of trophoblast-specific genes that influence maternal investment in offspring [14,15]. In one scenario, duplicated *PSG *genes are selected because they increase effective PSG dosage, thereby enhancing an effect on maternal investment in offspring. In this context, it is noteworthy that human PSG N domains contain putative integrin-binding 'RGD' motifs that are proposed to mediate cell interactions with the extracellular matrix [16,17] and immune cells [18]. Such PSG-mediated functions could potentially influence trophoblast invasion or maternal immune cell function. However, not all human, and none of the mouse, PSGs contain an RGD motif [7], suggesting that, if human RGD motifs are functionally significant, there has been diversification of function of some human, and all mouse, PSGs, relative to a putative RGD-containing ancestor. In the context of parent-offspring conflict, such divergence might reflect co-evolution of PSGs and their receptors, similar to the co-evolution of ligand / receptor pairs observed in host-pathogen interactions [19,20].

In this study, we sought to analyse PSG evolution to determine the extent and patterns of rodent and primate PSG sequence divergence by analysing intraspecific and interspecies DNA substitution rates in PSG coding regions. We also sought evidence in support of functionality of RGD and RGD-like tri-peptide motifs in PSG amino-terminal effector domains.

## Results

### Pairwise comparisons of all 4-domain mouse PSG with all 4-domain human PSG full-length amino acid sequences indicates conservation of the amino-terminal N domain

**Figure 1:**
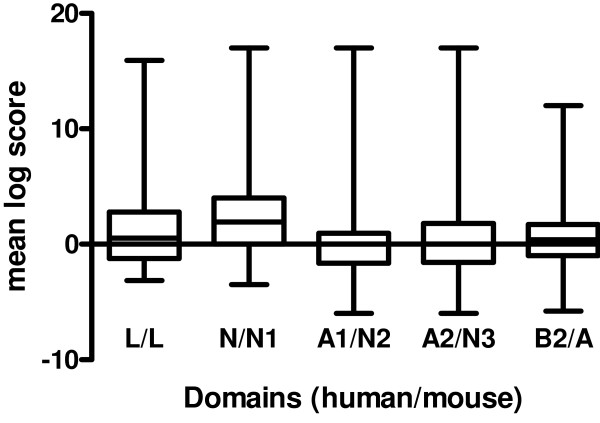
**Box and whisker plots for Dayhoff PAM 250 scores determined by ClustalX alignment of full-length mouse PSGs with full-length human PSGs. **At each position in the alignment, the Dayhoff PAM250 log score was determined for pairwise comparisons of each sequence in the set of mouse PSGs against all sequences in the set of human PSGs. Mouse Psg24, Psg30 and Psg31 along with human PSG2 and PSG5 were omitted from the analysis due to expansions or contractions of total domain complement which would complicate generation of the initial clustalX alignment. The scores were split into five groups, according to domain structure, and used to generate a box and whisker plot. Domain name abbreviations shown on the X-axis correspond to the following domain comparisons: L/L, human L versus mouse L domain; N/N1, human N versus mouse N1 domain; A1/N2, human A1 versus mouse N2 domain; A2/N3, human N2 versus mouse N3 domain; B2/A, human B2 versus mouse A domain. Significant differences of p < 0.0001 were observed for the N/N1 domain comparison when tested against the A1/N2 and A2/N3 data, and p < 0.0005 when tested against the B2/A data.

With the exception of mouse PSG24, PSG30 and PSG31 and human PSG2 and PSG5, all PSGs for which full length sequences are available have a structure based on four Ig-like domains and a leader sequence that is cleaved during post-translational processing. The only type of domain found in all rodent and primate PSGs is the N domain located at the amino terminus. Indeed, this domain is shared by all members of the extended CEA family, suggesting that it may contain important functional motifs. We sought to test this hypothesis with respect to PSG function, by analysing both full-length PSG sequences and selected domains of possible functional importance. Alignments of full-length 4-domain human and mouse PSG protein sequences were generated with ClustalX, followed by pairwise comparisons of all mouse sequences with all human sequences. Mean Dayhoff PAM250 log scores were calculated for each alignment position and grouped by domain. The scores within each of the four domains were then visualised using box and whisker plots (which show the median value, upper and lower quartiles plus range) (Fig. 1). The N domains exhibited significantly higher scores (p < 0.001) than the other three domains, with positive scores indicating conservation. There was no evidence of interspecies conservation of the other domains, which is unsurprising given the known lack of orthology between human A1 / mouse N2, human A2 / mouse N3, and human B2 / mouse A domain pairs.

### Novel rat PSG N1 domains identified by database searches

Rat N1 domain exon sequences were identified in NCBI and Ensembl databases. Three novel rat *PSG *genes were identified and named *PSG41, PSG42 *and *PSG43 *in keeping with accepted nomenclature [21]. We also identified a novel *PSG40 *splice variant with alternative leader and N1 domain exons, situated between the N1 and N2 domain exons of the published *PSG40 *sequence (NM_021677). Both BLAST and pattern matching methods retrieved the same rat *PSG *genes from different databases; therefore we considered our search to be exhaustive. All rat *PSG *genes were found to reside on contig NW_047556 and this was used for the prediction of remaining exons for each *PSG *gene based on BLAST generated alignments with mouse *Psg *gene sequences (Table 1). The CDS sequences of the novel predicted rat *PSG *genes and *PSG40 *splice variant are listed in additional file 1. We used our predicted sequences in preference to the publicly available sequences in our analyses.

### SplitsTree analysis reveals relatively high contradiction in rat PSG N1 domain alignments, compared to mouse

Following the preliminary identification of amino-terminal N domain conservation, we planned to use an evolutionary tree building approach to further examine inter-domain relationships in rodent and primate *PSG*s. However, using split decomposition analysis, McLenachan et al. [22], in their study of a subset of human *PSG*s, concluded that it is not possible to accurately determine branch points in an evolutionary tree of human *PSG*s. Split decomposition analysis identifies contradictory relationships within alignment data; for example, there may be a pattern grouping *PSGX *and *PSGY *together, and another pattern grouping *PSGY *and *PSGZ *together [23]. This information is normally approximated when drawing evolutionary trees, however split decomposition is a non-approximation method that permits the building of trees with support indicated for relationships based on all patterns in the data. Such analysis can therefore predict to a limited extent the occurrence of sequence homogenisation e.g. by gene conversion or positive selection.

**Figure 2:**
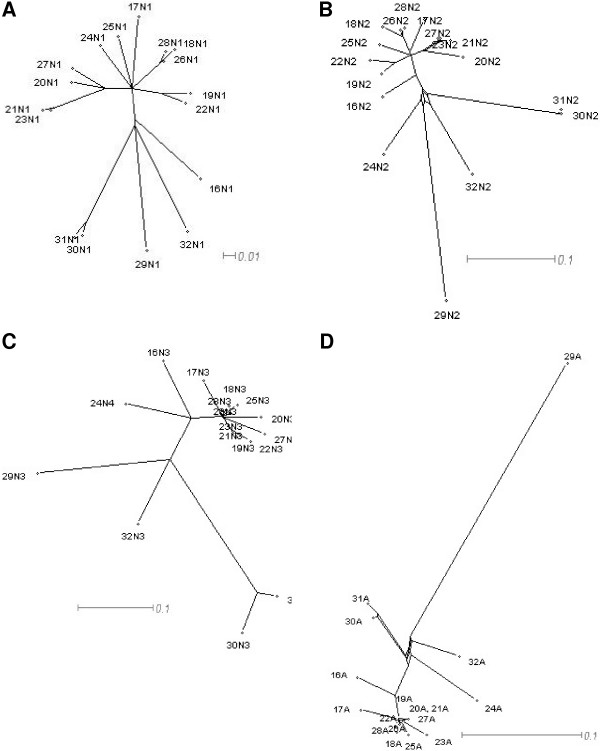
**Split decomposition graphs for all mouse Psg domain exons and rat PSG N1 domain exons for observed (Hamming) distances. **Split decomposition analysis was performed using nucleotide sequences for individual groups of *PSG *domain exons. (A) N1 domain exons; (B) N2 domain exons; (C) N3 domain exons (the N4 domain exon of *Psg24 *is used instead of N3; see Fig. 3B in McLellan et al. [41] for explanation); (D) A domain exons. Numbers indicate respective *PSG *genes. Scale bars represent 0.01 (A) or 0.1 nucleotide substitutions per site (B, C, D).

**Figure 3:**
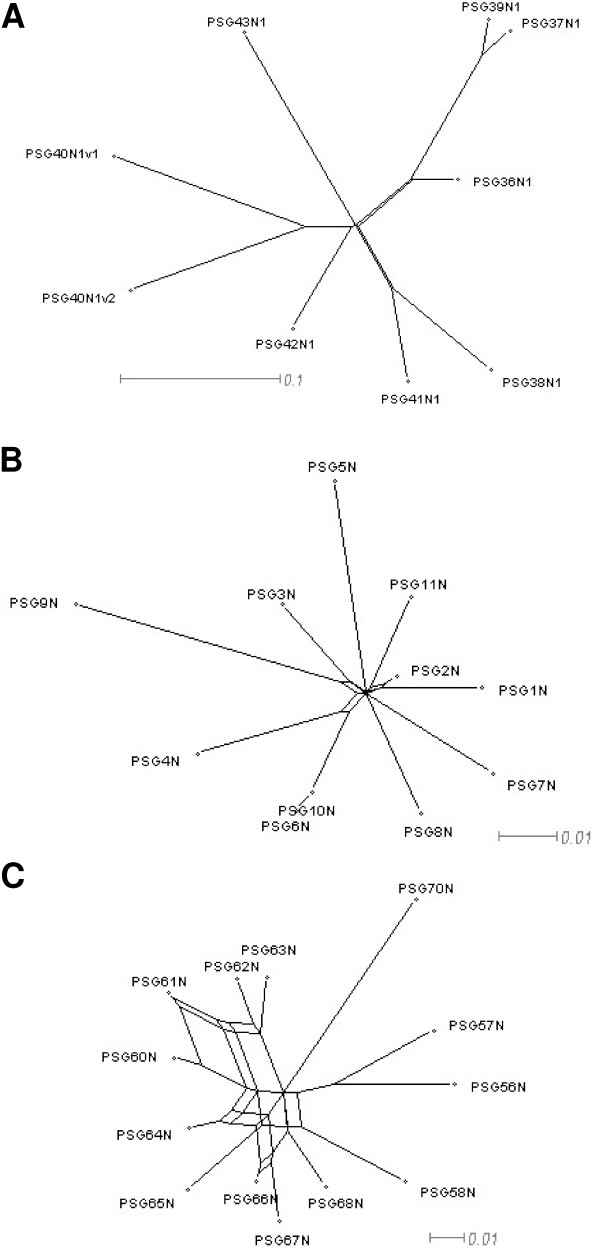
**Split decomposition graphs for the rat PSG N1 domain exons, human PSG N domain exons, and baboon PSG N domain exons for observed (Hamming) distances. **Split decomposition analysis was performed using nucleotide sequences for individual groups of *PSG *domain exons. (A) rat N1 domain exons; (B) human N domain exons; (C) baboon N domain exons. Scale bars represent 0.01 nucleotide substitutions per site. In (A) the putative N1 domain exon splice variants of *PSG40 *are identified with the suffix 'v1' for the published variant (NM_021677) and 'v2' for our predicted variant.

We performed split decomposition analysis on nucleotide sequences using the SplitsTree4 program [24] on the individual domain exons of mouse *Psg *genes (Fig. 2). For a more complete analysis of N1 domains we also performed the analysis using rat N1 domain exons, all known human N1 domain exons and all known baboon N1 domain exons (Fig. 3). We detected no conflicting signals for mouse *Psg *N1 domain exons (Fig. 2A), in contrast to the human N domain exons (Fig. 2B). However, our results for human N1 domains (Fig. 3B) differ from those obtained by McLenachan et al. [22] because we observed only two contradictions: *i*. regarding the relationship of *PSG4 *and *PSG9 *to each other, and to their nearest neighbours *PSG3 *and the common ancestor of *PSG6 *and *PSG10 *and, *ii*. between 'the relationship of PSG2 to PSG1 and PSG11'. This discrepancy is probably due to our inclusion of four extra *PSG *N1 domain sequences, and the fact that the *PSG11 *sequence (GenBank: M69025) used by McLenachan et al. [22] has been updated.

Analysis of the mouse N2 domains indicates numerous contradictions in the alignments of the *Psg24*, *Psg29*, *Psg30*, *Psg31 *and *Psg32 *group (Fig. 2B). In contrast, the N3 domains exhibit no discernable conflicts (Fig. 2C). The A domain only showed contradiction within the *Psg24*, *Psg29*, *Psg30*, *Psg31 *and *Psg32 *group (Fig. 2D). Examination of the rat *PSG *N1 domain exon alignments demonstrated minor contradictions between the common ancestor of *PSG36*, *PSG37 *and *PSG39 *and that of *PSG38 *and *PSG41 *(Fig. 3A). In contrast to all the other *PSG *N1 domains thus compared, the baboon *PSG*s demonstrate considerable conflicting signals as demonstrated by the 'spider's web' appearance of the SplitsTree graph (Fig. 3C).

### Phylogenetic analysis indicates interspecific amino-terminal N domain conservation and identifies potential mouse / rat orthologues

**Figure 4:**
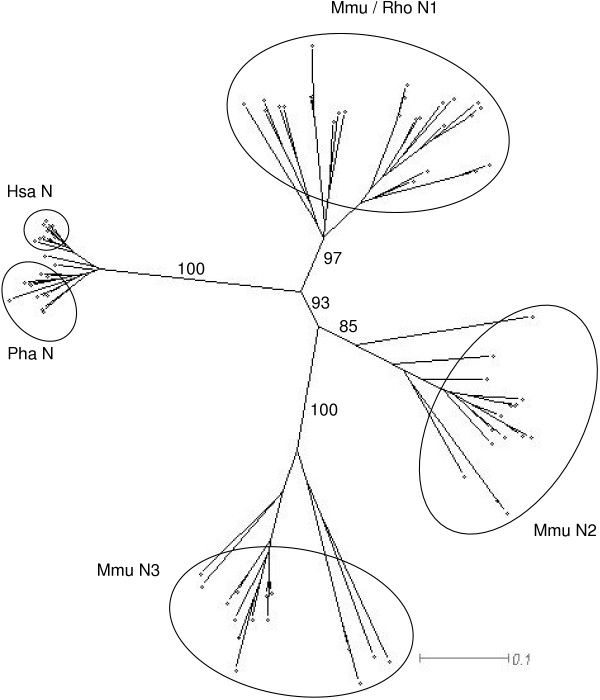
**Phylogeny of the mouse N1, N2 and N3 domains, rat N1 domains and human N domains. **NJ-tree of N domain nucleotide sequences on ClustalX alignments of corresponding amino acid sequences showing the evolution of mouse (Mmu) PSG N1, N2 and N3 domains in comparison with rat (Rho) N1 domains, human (Hsa) N domains and baboon (Pha) N domains. Alignments were bootstrapped 1000 times yielding the values shown for the main branches. The scale bar represents 0.1 nucleotide substitutions per site.

**Figure 5:**
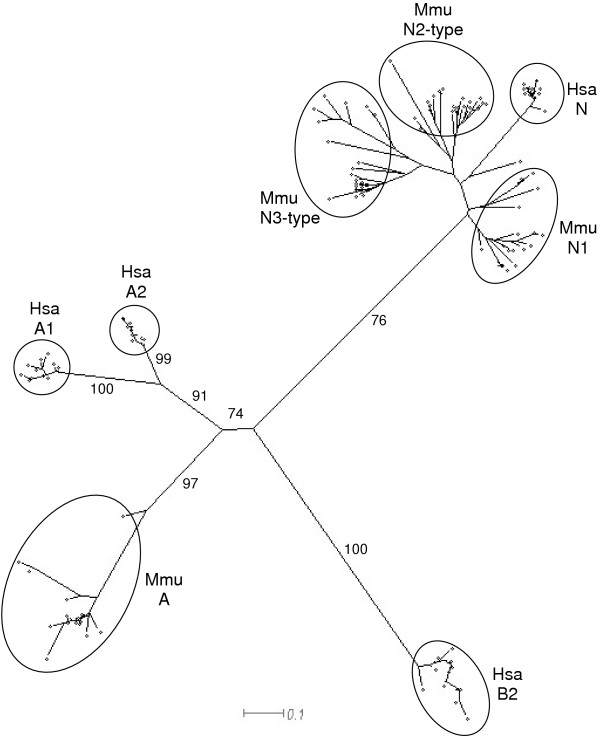
**Phylogeny of all known mouse and human PSG N, A and B domains. **NJ-tree of mouse (Mmu) and human (Hsa) N, A and B domain nucleotide sequences on ClustalX alignments of corresponding amino acid sequences showing the evolutionary relationships between domain types. Alignments were bootstrapped 1000 times yielding the values shown on the major branches. Scale bar represents 0.1 nucleotide substitutions per site.

Few examples of orthologous relationships between *PSG *sequences have been identified. In order to compare the relationship between rodent and primate amino-terminal N domain exon coding sequences, an NJ tree was produced (Fig. 4). The tree was generated from ClustalX alignments of nucleotide sequences, with bootstrapping 1000 times to test the reliability of branches. The human and baboon N sequences formed one distinct cluster, the mouse and rat N1 sequences formed a second, the mouse N2 domains formed a third and the mouse N3 domains formed a fourth. Of particular interest was the split between the ancestral N-type domain and the common ancestor of the N2 and N3 domains. The confidence of this split was 93% and demonstrates that the mouse N1 domains are more closely related to primate N domains than to the mouse N2 and N3 domains. A similar comparison of the entire set of mouse and human *PSG *domains confirmed that the interspecific N domain clustering is unique because the human *PSG *A1 and A2 domains segregated into distinct branches (sharing a common ancestor with the mouse A domains) and the B2 domains cluster on a distinct branch (Fig. 5).

**Figure 6:**
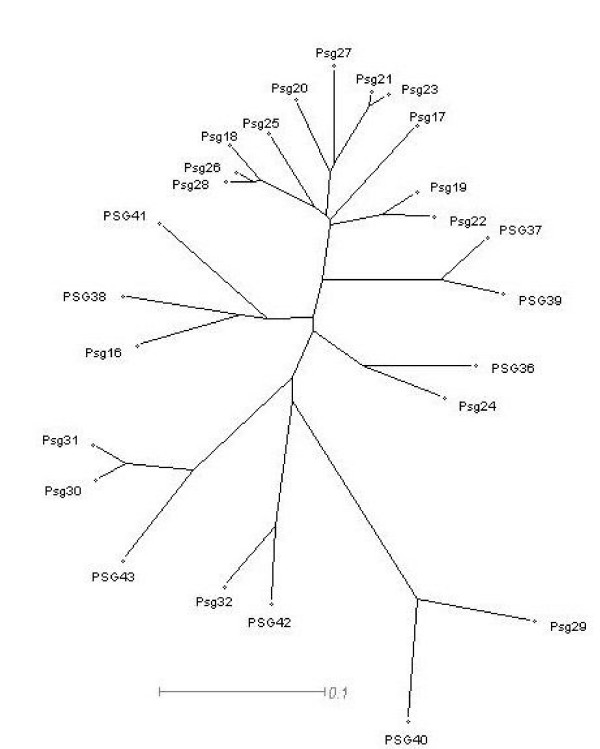
**NJ tree of alignments of complete CDS of all known mouse and rat PSGs. **Sequences of *PSG40 *– *PSG43 *are *de novo *predictions. Data were bootstrapped 1000 times and all major branches yielded values of 95–100%. The scale bar represents 0.1 nucleotide substitutions per site.

Mouse and rat *PSG *gene coding sequences were analysed using an NJ plot which highlighted four putative orthologous relationships, as follows: rat *PSG36 *and mouse *Psg24*; rat *PSG40 *and mouse *Psg29*; rat *PSG42 *and mouse *Psg32*; rat *PSG38 *and mouse *Psg16 *(Fig. 6). There is also distinct branching of rat *PSG43 *with mouse *Psg30 *and *Psg31*. The orthologous relationship is also supported for *PSG36 *and *Psg24 *because both contain five N domains.

### PSG N domain sequences are generally conserved but alignments reveal specific regions that may be diverging

**Figure 7:**
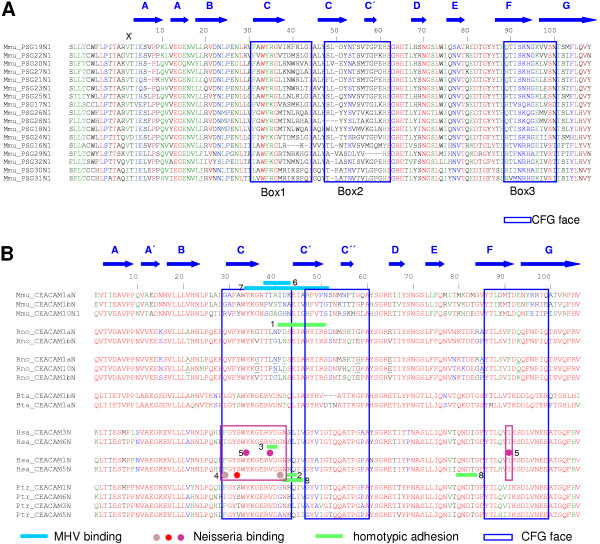
**High amino acid sequence variability is found in the CFG faces of mouse PSG N1 domains. **Alignments of the mouse PSG N1 domain amino acid sequences were performed using ClustalW. The locations of the β-strands (A-G) were derived from the crystal structure of the mouse CEACAM1 N domain [25], and are indicated by blue arrows. The boxed amino acids sequences form the CFG face of the N domain (deduced by structural modelling). (A) Alignment of mouse PSG N1 domain amino acid sequences. The signal peptide (leader) cleavage site is shown as a dotted line and N domain amino acid numbering commences from the first amino acid of the mature N domain. (B) Alignment of CEACAM N domains (minus signal sequences) showing all N domain interactions with pathogens and known binding partners (referenced as follows: 1 [48]; 2 [49]; 3 [50]; 4 [51]; 5 [52]; 6 [53]; 7 [54]; 8 [55]).

**Figure 8:**
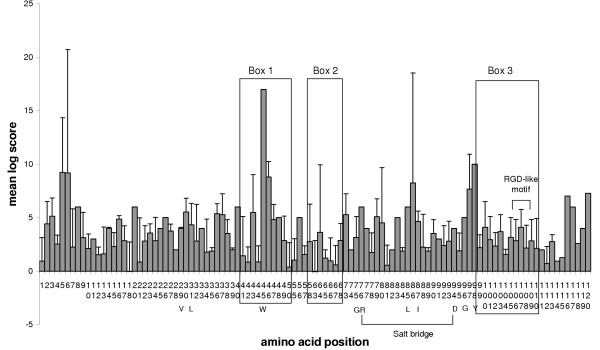
**Dayhoff PAM250 plot for ClustalX-aligned mouse N1 domain amino acid sequence comparisons. **At each position in the alignment, the Dayhoff PAM250 log score was determined for pairwise comparisons of each sequence in the set against all the others in the set. Mean and standard deviation were calculated for scores at each residue position. Regions representing the CFG face are boxed (1–3) and an RGD-like motif is indicated. Other specified amino acids are denoted by the single letter code. Note that amino acid positions are numbered in vertical orientation.

**Figure 9:**
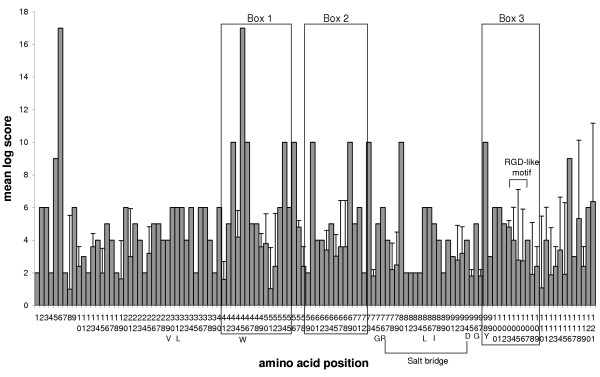
**Dayhoff PAM250 plot for ClustalX-aligned human N domain amino acid sequence comparisons. **At each position in the alignment, the Dayhoff PAM250 log score was determined for pairwise comparisons of each sequence in the set against all the others in the set. Mean and standard deviation were calculated for scores at each residue position. Regions representing the CFG face are boxed (1–3) and an RGD-like motif is indicated. Other specified amino acids are denoted by the single letter code. Note that amino acid positions are numbered in vertical orientation.

**Figure 10:**
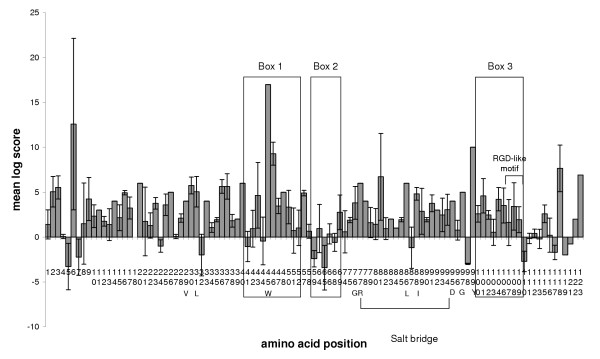
**Dayhoff PAM250 plots for ClustalX-aligned N1 (mouse) and N (human) domain amino acid sequence comparisons. **At each position in the alignment, the Dayhoff PAM250 log score was determined for pairwise comparisons of each sequence in the mouse set against all sequences in the human set. Mean and standard deviation were calculated for scores at each residue position. Regions representing the CFG face are boxed (1–3) and an RGD-like motif is indicated. Other specified amino acids are denoted by the single letter code. Note that amino acid positions are numbered in vertical orientation.

The crystal structure of mouse CEACAM1 (soluble murine sCEACAM1a [1,4]) has been resolved [25]. Comparison of the mouse PSG N1 domains identifies the predicted β-sheet-forming CFG β-strands as the most variable regions of the N domains (Fig. 7A). The CFG face of CEACAM N domains has been shown to interact with pathogens and mammalian proteins (Fig. 7B). Within Box 1 and Box 2, there is considerable variation between mouse N1 domains, which is illustrated quantitatively using Dayhoff charts (Figs. 8 – 10). Positive Dayhoff scores and generally low standard deviations indicate good conservation of mouse PSG N1 domains (Fig. 8), and even stronger conservation of human PSG N domains (Fig. 9). The latter may be explained by homogenisation of human *PSG *gene sequences [22]. Dayhoff score analysis using comparisons of all mouse N1 domain versus all human N domain ClustalX aligned sequences gives an indication, at the amino acid level, of the general pattern of evolution of these domains since the rodent / primate divergence (Fig. 10). Again, the majority of residues exhibit good conservation, and relatively little variability is observed between pair-wise comparisons particularly with regard to residues that are involved in protein folding. The reduction in size of Box 2 in Fig. 8 and Fig. 10 is explained by deletions of mouse DNA sequences, requiring exclusion of the corresponding amino acids from the analysis.

**Figure 11:**
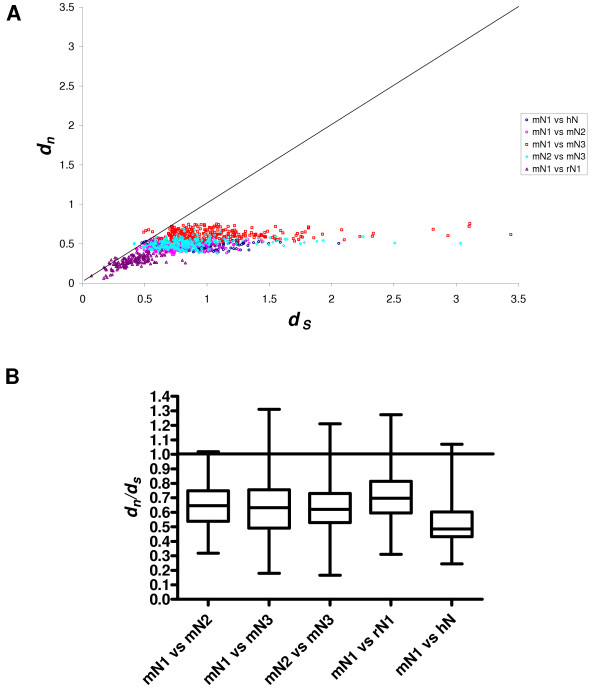
**Nonsynonymous versus synonymous substitution rates for pairwise comparisons between N domains. **The number of nonsynonymous substitutions per nonsynonymous site (*d*_n_) and the number of synonymous substitutions per synonymous site (*d*_s_) was calculated using the method of Yang and Neilson [46] for pairwise nucleotide comparisons. The N1, N2 and N3 domains of mouse PSGs (mN1, mN2 and mN3, respectively), the N1 domain of rat PSGs (rN1) and the N domain of human PSGs (hN) comprised individual data sets that were analysed in the following comparisons: mN1 vs mN2; mN1 vs mN3; mN2 vs mN3; mN1 vs rN1; mN1 vs hN. (A) Plot of *d*_*n *_against *d*_*s *_where each data point represents a pairwise comparison of a nucleotide sequence taken from each set under comparison. The 45° line of equivalence is drawn where *d*_*n *_= *d*_*s*_. (B) Box and whisker plot of *d*_*n*_/*d*_*s *_calculated from the pairwise comparisons of all sequences in one dataset against all sequences in the other dataset. Significant differences of p < 0.0001 (calculated by the Mann-Whitney method) were observed between all comparisons except intra-mouse comparisons.

To gain further insight into mouse *Psg *N domain exon evolution, the N1, N2 and N3 domain exons of mouse *Psg *genes (mN1, mN2 and mN3, respectively), the N1 domain exons of rat *PSG *genes (rN1) and the N domain exons of human *PSG *genes (hN) were analysed in the following comparisons: mN1 vs mN2; mN1 vs mN3; mN2 vs mN3; mN1 vs rN1; mN1 vs hN. Synonymous (*d*_s_) and non-synonymous (*d*_n_) substitutions per synonymous and non-synonymous site, respectively, were determined in each case for all combinations of *PSG *gene pairwise comparisons, and box and whisker plots were generated from the data (Fig. 11). The majority of data points derived from individual comparisons lie under the 45° line of equivalence where *d*_n _= *d*_s_, and most variation in the comparisons lies within the values of *d*_s _(Fig. 11A). When the data are presented as box and whisker plots, the values are indicative of conservation, with median values ranging from 0.48 – 0.70 (Fig. 11B). The higher values for median *d*_n_/*d*_s _in the mN1 vs rN1 comparison appear to be the result of a tighter *d*_s _distribution as observed in Fig. 11A, with values not exceeding one substitution per synonymous site in any pairwise comparison.

**Figure 12:**
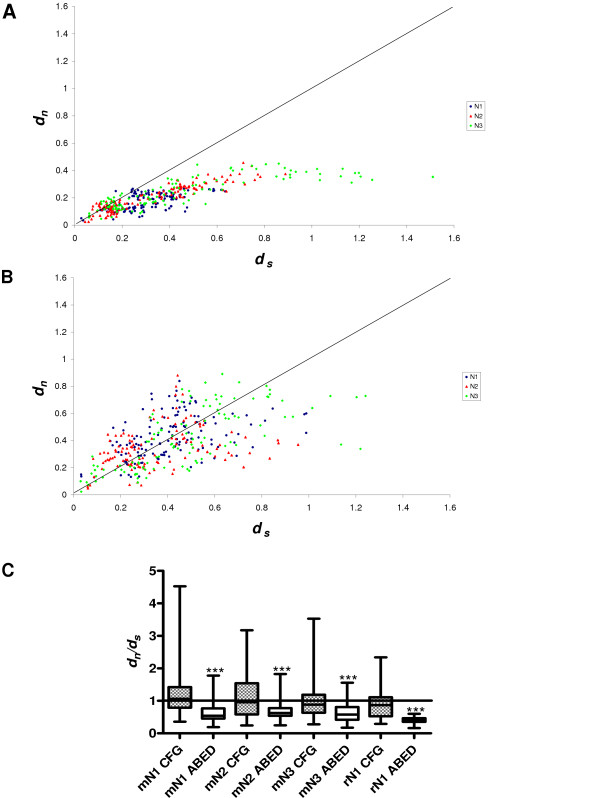
**Nonsynonymous versus synonymous substitution rates in the mouse N domain CFG and ABED faces. **The nucleotide sequences encoding the CFG and ABED faces of the N1 domain and equivalent regions of the N2 and N3 domains were separated and compared individually. The number of nonsynonymous substitutions per nonsynonymous site (*d*_*n*_) and the number of synonymous substitutions per synonymous site (*d*_*s*_) were calculated using the method of Yang and Neilson [46] for pairwise nucleotide comparisons. Plots are shown of *d*_*n *_versus *d*_*s *_for regions comprising (A) the ABED face and, (B) the CFG face, where each data point represents a pairwise comparison of two nucleotide sequences taken from the dataset being examined. The 45° line of equivalence is drawn where *d*_*n *_= *d*_*s*_. (C) Box and whisker plot of *d*_*n*_/*d*_*s *_calculated from pairwise comparisons of all sequences in one dataset against all others in the set. Data derived from sets of rat N1 CFG and ABED faces were also analysed for comparative purposes. Significant differences between CFG and ABED faces for each domain are shown, where '***' is p < 0.0001 (calculated by the Mann-Whitney method).

In view of the sequence variations in the CFG face, which are visible in alignments (Fig. 7A), against a background of overall conservation, as estimated from *d*_n_/*d*_s _analysis, we sought to determine whether the *d*_n_/*d*_s _values were higher in the CFG face than the ABED face of the N1 domain. Nucleotide sequence alignments were generated using all mouse *Psg *N1 domain exons (based on protein alignments), and the nucleotides present in the three sections comprising the CFG face (Boxes 1, 2 & 3; Fig. 7A) were separated from those comprising the ABED face. The two new sets of data were analysed individually to determine mean *d*_n _and *d*_s _values from pairwise comparisons of all sequences within each dataset (Fig. 12). A plot of *d*_n _vs *d*_s _for the ABED face of the mouse N1, N2 and N3 domains (Fig. 12A) demonstrates a distribution of pairwise-alignment data points which overwhelmingly lie below the line of equivalence. However, a similar plot generated from analysis of the CFG face has data points distributed approximately equally on both sides of the line of equivalence (Fig. 12B). This is due predominately to a higher number of non-synonymous substitutions. The values of *d*_n_/*d*_s _obtained for the CFG face in the N1, N2 and N3 domains of the mouse and the N1 domain of the rat are all significantly greater than the values obtained for the ABED face (p < 0.0001, Fig. 12C). The *d*_n_/*d*_s _values obtained for the mouse N1, N2 and N3 domain CFG faces equal or exceed 1.0, with the highest median value of 1.1 observed in the N1 domain. The rat N1 domains are more conserved, with *d*_n_/*d*_s _values derived from both the CFG and ABED faces under 1.0 on average.

### Evidence of conservation of RGD-like motifs in mouse N1 domains

**Figure 13:**
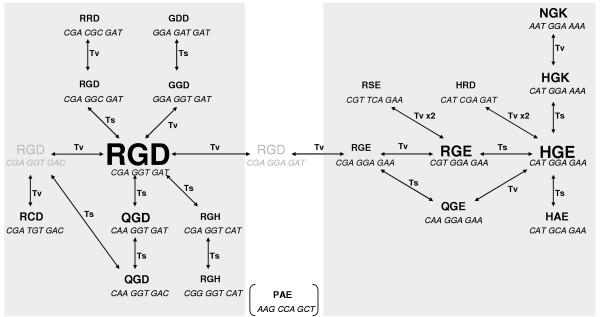
**Relationship between RGD-like motifs in human, baboon, mouse and rat PSG N domains**. The font size used for each tri-peptide motif represents relative abundance among the PSG proteins, and the codon sequences are shown underneath. Arrows represent single or double (x2) transitions (ts) or transversions (tv) as indicated. Motifs and codon sequences in grey type are intermediates that have not been observed *in vivo*. Primate RGD-like motifs cluster naturally in the left-hand box, whereas those of the rodents cluster in the right-hand box. The baboon derived PAE motif is an outlier and is bracketed.

Within Box 3 of the CFG face (Fig. 7A) there is evidence of conservation of putative integrin-interacting RGD-like motifs in the mouse N1 domain, which may have functional significance. To investigate this possibility further, a survey of all mouse, rat, baboon and human PSG RGD, and related, motifs was compiled (Fig. 13). Extant primate and rodent PSG RGD-like motifs are linked in sequence space by an RGD motif encoded by the sequence CGA GGA GAT which, incidentally, is not observed in any of the extant *PSG *coding sequences. The most commonly observed motif, RGD, is encoded by CGA GGT GAT, and the majority of variants are closely related to this sequence. In rodents, RGE and HGE are the most commonly observed motifs. However, the NGK motif, which is not an RGD-like motif as we have defined it, is well represented, and is separated in sequence space from HGE by a transition and a transversion.

Of the seventeen aligned mouse PSG N1 domain exon sequences, 53% possess a tri-peptide at the site of the RGD-like motif belonging to the RGD-like 5-1-4 tri-group (as defined in the Methods section). For comparative purposes, tri-groups were determined for tri-peptide motifs at fifty random positions within the alignment. The number of most commonly represented tri-groups at each position was expressed as a percentage of the number of aligned sequences, and the mean and standard deviation was determined to indicate the mean maximal tri-group representation for the 50 random alignment positions. The control value obtained was 67.6 ± 22.9%; the value of 53% of 5-1-4 tri-groups at the RGD site therefore lies within the control range, albeit 14.6% below the mean value. However, a more revealing statistic is derived from aligning the mouse N1 domains with the mouse N2 and N3 domains (see additional file 2), compared to aligning the mouse N1 domains with the human N domains. In the former comparison (mouse N1 vs N2 and N3 domains) the most commonly represented tri-group is 4-2-5, with 27% representation. This tri-group is not RGD-like and its representation is lower than the mean maximal tri-group representation of 49.8 ± 22.7% determined for fifty random alignment positions. However, when the mouse N1 domain is aligned with the human N domain, the most commonly represented tri-group is the RGD-like 5-1-4 group which has 59% representation, comparable to the mean maximal tri-group representation of 60.7 ± 20.4%.

## Discussion

We recently collated the full-length coding sequences of the entire mouse *Psg *gene family [7]. In the present study we aimed to identify evolutionary signals embedded in *Psg *gene and PSG protein sequences to determine whether PSG protein function has diverged between the rodent and primate lineages, and to attempt to understand the reasons for the independent expansions of rodent and primate *PSG *gene families.

Mouse and human PSG protein amino-terminal N domains exhibit different patterns of evolution. McLenachan et al. [22] analysed the evolution of a subset of human PSGs using split decomposition analysis and found, in individual comparisons of N, A1, B2 and C domain exons, strong contradictions in alignments, which they suggested was due to gene conversion and/or positive selection. Our similar analysis of an expanded set of human *PSG *sequences revealed a detectable, but less marked, degree of homogenisation. Analysis of mouse N and A domain exons showed that, in general, there is less evidence of purifying selection compared to the human, although there are examples of gene conversions as described previously for the closely related *Psg21 *and *Psg23 *genes [12]. Detailed analysis of alignments using plots of Dayhoff scores confirmed the difference between mouse and human N domain evolution.

Using *d*_n_/*d*_s _analysis for interspecies comparisons, we found that the PSG protein amino-terminal N and N1 domains are relatively conserved, consistent with conservation of function in rodents and primates. However, inspection of mouse PSG N1 domain alignments, and scrutiny of corresponding Dayhoff scores, revealed regions of apparently poor conservation. These regions correspond to the CFG face within the N1 domain of CEACAM1. In the CEACAM family, the CFG face interacts with pathogens and mammalian proteins. Comparisons of *d*_n_/*d*_s _values obtained from the CFG and ABED faces of mouse N1, N2 and N3 domains confirmed that the CFG face has evolved more rapidly than the ABED face in all three domains. The greatest effect was observed in the N1 domain exon with a doubling of the *d*_n_/*d*_s _ratio in the CFG face compared with the ABED face. The *d*_n_/*d*_s _ratio of 1.1 suggests weak positive selection on the CFG face of the N1 domain. The increase in the *d*_n_/*d*_s _ratio appears to be mainly due to an increase in the *d*_n _value, indicative of diversification. The high d_n_/d_s _values for the CFG face in the N2 and N3 domains, which are not known to interact with ligands, could be due to a low contribution of these sequences to the structural integrity of the IgV-like domain.

Interestingly, the rat N1 domain CFG face does not appear to have evolved as rapidly as the mouse N1 domain, with a *d*_n_/*d*_s _ratio of 0.9. This observation, combined with the relatively smaller number of *PSG *genes identified in the rat (eight to date, compared to seventeen in the mouse) and the higher level of gene homogenisation implied by split decomposition analysis suggests that the rat *PSG *gene family has not expanded or diversified as extensively as the mouse. However, we cannot exclude the possibility that further rat *PSG *genes may yet be identified because there may be under-representation in the WGS database [26]. Notwithstanding this possibility, there has clearly been ongoing turnover of the *PSG *gene family in all of the lineages analysed, as there are no known human orthologues of rat and mouse *PSG*s, and only four potential orthologous relationships between known rat and mouse *PSG*s.

These findings suggest partial conservation of PSG N domain function across rodent and primate lineages. However, the relaxed constraint on the CFG face of mouse PSGs suggests diversification of binding partners or modification of existing ligand-binding kinetics, analogous to the CEACAMs. This observation receives experimental support from the recent observation that treatment of mouse macrophages *in vitro *with recombinant mouse PSG17N, or human PSG1 or PSG11, induces cytokine expression; however, only in the case of mouse PSG17N does this depend on CD9 receptor expression [27]. Divergence of PSG function is also suggested by differences in the level and developmental timing of expression of different mouse PSGs [7,12], expansion of N domain number in PSG24, PSG30 and PSG31 [7], and loss of secretory signals in PSG32 and in the brain-specific splice variant of PSG16.

As noted above, the only PSG receptor identified to date is the integrin-associated tetraspanin, CD9, which binds the N1 domain of mouse PSG17 but not, apparently, to human PSGs [28]. However, a peptide containing the RGD motif from the human PSG9 N domain binds to a receptor on a promonocytic cell line suggesting that some human PSGs may effect their functions through an integrin-type receptor [18]. In this context, the high frequency of the RGD motif on an exposed loop in primate PSG N domains (seven of ten in human and five of fifteen in baboon) may be significant. Rodent PSG N1 domains do not have an RGD motif, but have a high frequency of the RGD-like motifs RGE, HGE and HAE on the CFG face. Under the null hypothesis that these motifs are unlikely to underpin structural integrity of the N1 domain and are therefore free of constraint, our analysis reveals evidence of unexpected conservation of RGD-like motifs in the N1 domain, which have been lost in the N2 and N3 domains. Given the high transition and transversion rates in the N1 domain and the fact that the mouse N1, N2 and N3 domains share a common ancestor after the divergence of the rodent / primate lineages, the conservation of RGD-like motifs exclusively in the N1 domain may have functional significance. We note that the RGE motif in the context of the POEM protein induced apoptosis of MC3T3-E1 cells *in vitro *[29]. We speculate that certain RGE or RGE-like motifs may elicit weak cell attachment, followed by apoptosis – a combination of properties, reminiscent of snake venom disintegrins [30,31], that could have important functional implications in the context of the extensive tissue remodelling that occurs during placentation [32].

In summary, our data are consistent with experimental evidence indicating functional convergence of rodent and primate PSGs, in spite of the independent expansions of the gene families in the two lineages. In the context of parent-offspring conflict, the homogenisation of human PSG sequences is consistent with the theory that placental hormones encoded by multigene families are monofunctional and selected for high expression, possibly due to coevolution with physiologically conflicting maternal mechanisms [15]. However, the evidence for positive selection on the CFG face of the N1 domain implies divergent evolution of rodent PSGs. Allied to the evidence for functionality of putative integrin-interacting RGD-like motifs in rodents, a scenario can be envisaged whereby the different RGD-like motifs observed in human and baboon PSGs also suggest some degree of functional divergence in these species.

## Conclusion

Our analysis provides evidence for conservation of rodent and primate PSG amino-terminal N domains, with ongoing independent expansion of the gene families in the two lineages. There has been some diversification of the CFG face of mouse N1 domains, a region that includes putative integrin-interacting RGD-like motifs. Our analysis provides reassurance that the mouse *Psg *gene family is a suitable model system for the analysis of human *PSG *gene function.

## Methods

Perl programs were written to perform most general sequence manipulations and iterative tasks and executed under ActivePerl v5.8.3 [33] on a Windows 2000 (Microsoft) platform.

### Identification of novel rat PSG N1 domain exons

Blast searches of the NCBI [34] and Ensembl [35] RGSC3.1 rat genome databases were performed using coding sequences from known rat PSGs (*PSG36*-*PSG40*) and mouse PSGs. Additionally, a search pattern was developed and used to interrogate the Rattus_norvegicus.RGSC3.1.nov.dna_rm.contig.fa.gz archive obtained from the Ensembl FTP resource [36]. The search pattern was derived manually from alignments of amino acid sequences from the N domain exon of all known mouse and rat *PSG*s (mouse *PSG16*-*PSG32 *and rat *PSG36*-*PSG40*) generated using the ClustalX 1.81 windows interface [37]. In PROSITE format [38] the search pattern used was S-x-R-E-x(5)-G-x(3)-[IL]-x(3)-T-x(2)D-x(3)-Y-x(17,18)-L-x-V. Analysis was performed essentially as described [39], with the program modified to search for the selected pattern in peptides of fifty amino acids or greater derived from genomic DNA sequences translated in all six open reading frames. ClustalX alignments were produced using the complete open reading frames returned by the program combined with the N1 domains of rat PSG36-PSG40. The alignments were trimmed to include only N1 domain exon sequence and a Neighbour-Joining tree was generated using MEGA version 2.1 software [40] to aid the identification of the new sequences.

### Phylogenic analysis

Mouse PSG sequences were obtained from McLellan et al. [41], rat PSG sequences were obtained as described above, human PSG sequences were obtained by name searches at the NCBI Entrez (nucleotide or protein options) database [42] and baboon N1 domain sequences were obtained as described [43]. To generate protein alignments for examination by eye, a Web based ClustalW utility was used [44], otherwise protein sequences were aligned with the ClustalX using the default parameters. Nucleotide alignments were generated based on ClustalX protein alignments, such that where a single dash was placed in the amino acid alignment, three dashes were placed in the equivalent codon position in the nucleotide alignment. The nucleotide alignments were then analysed using SplitsTree version 4b [24] and software and NJ trees were generated from the data (with bootstrapping 1000 times to test the reliability of branches). Individual domains of the mouse PSGs were also analysed by the split decomposition method using the same software. During NJ or Splitstree tree-building, the Jukes-Cantor [45] correction for multiple hits was applied and positions with gaps were ignored.

### Comparisons of amino acids encoded at each site within alignments

Multiple alignments of either one set (e.g. all mouse PSG N1 domain exons only) or two sets (e.g. all mouse PSG N1 and N2 domain exons) of amino acid sequences were produced using ClustalX. A Perl program was written to perform the subsequent analysis. At each position of the alignment, the Dayhoff PAM250 log score was determined for pairwise comparisons of each sequence in the set against all the others in the set in one-set analyses, or of all set 1 sequences against all set 2 sequences in two-set analyses. The mean and standard deviation of scores obtained for the pairwise comparisons at each site were determined to give an indication of the general level of conservation and variability at the site. Sites where gaps were present in any of the sequences were not analysed. Where full-length mouse and human PSG amino acid sequences were compared, the scores were split into five groups at domain junctions and a box and whisker plot produced.

### Evolutionary analysis

ClustalX was used to produce multiple alignments of either one set of amino acid sequences (e.g. all mouse PSG N1 domain exons only) or two sets combined (e.g. all mouse PSG N1 and N2 domain exons). These alignments were used to inform the alignment of corresponding nucleotide sequences as described above. Values of *d*_s _and *d*_n _were determined for pairwise comparisons of each sequence in a set against all the others in the set for one-set analysis, or of all set 1 sequences against all set 2 sequences for two-set analysis. The analysis was performed according the method of Yang and Neilsen [46] using the 'YN00' program in the PAML3.14 software package [47]. Before each pairwise comparison was executed, pairs of aligned sequences were extracted from the alignment file, placed in a Phylip format file and gapped positions were removed. Plots of *d*_*n *_vs *d*_*s*_, and box and whisker plots of *d*_*n*_/*d*_*s *_were produced in order to visualise the data. Where statistical significance was evaluated, the Mann-Whitney test was applied.

### Analysis of tri-peptide amino acid property groupings

A perl program was written to analyse ClustalX alignments of mouse and human PSG N domain exons. These alignments were inspected and modified where necessary. For a tri-peptide at a given position within an alignment, a tri-group code was generated for tri-peptide motifs based on amino acid properties of the residues in the motif where group 1 contains G, A, S, T; group 2: V, L, I, M; group 3: F, Y, W; group 4: D, N, E, Q; group 5: H, K, R; group 6: P; group 7: C. For example, an RGD tri-peptide motif is represented by tri-group code 5-1-4 as arginine is in group 5, glycine is in group 1, and aspartate is in group 4. Conversely, tri-group 5-1-4 is 'RGD-like' in terms of the biochemical properties of the constituent amino acids. The number of sequences in the alignment containing each group code at a given position was determined. The most highly represented group code in the alignment at that position was used in the analysis. The program was designed to compare a user selected tri-peptide motif position with fifty randomly selected tri-peptide motif positions.

## Authors' contributions

A. McLellan performed data collection and analysis and co-wrote the manuscript. W. Zimmermann and T. Moore co-conceived the project and co-wrote the manuscript.

**Table 1:** Rat *PSG *genes: nomenclature and references. Previously and newly identified rat *PSG *genes are listed with GenBank references. Where the GNOMON predicted sequence in GenBank differs from our prediction this is denoted by a single asterix beside the nucleotide accession number. A double asterix indicates the prediction of a putative splice variant with an alternative leader and N1-domain exon.

**gene name**	**alternative/ old name**	**accession number (nucleotide)**	**accession number (protein)**	**NW_047566.1 contig (CDS start and end positions and orientation)**	**Notes**
*PSG36*	*CGM1*	NM_012702	XP_218391	947162 – 958811 (F)	
*PSG37*	*CGM3*	NM_019126	NP_061999	1543892 – 1552811 (R)	
*PSG38*	similar to brain *CEA*	XM_214842*	XP_214842	859024 – 870967 (F)	
*PSG39*	*CGM6*	XM_218398	XP_218398	1562043 – 1571612 (R)	
*PSG40*	*CGM8*	NM_021677**	NP_067709	1009499 – 1015560 (F)	putative novel splice variant
*PSG41*		XM_218390*	XP_218390	888980 – 904807 (F)	
*PSG42*				1108370 – 1118331 (F)	
*PSG43*				1149404 – 1185428 (F)	

## Supplementary Material

Additional File 1 An ASCII text file containing the CDS sequences of novel predicted rat *PSG41*, *PSG42 *and *PSG43 *and a novel splice variant of *PSG40*. Click here for file

Additional File 2 A rich text format file containing the Clustal W amino acid sequence multialignment of PSG N1, N2 and N3 domains. The RGD-like motif is boxed for comparison between domains. Click here for file
