# Supplementary material for: Conservation of pregnancy-specific glycoprotein (PSG) N domains following independent expansions of the gene families in rodents and primates
Source: BMC Evol Biol. 2005 Jun 29;5:39. doi: 10.1186/1471-2148-5-39 (PMC1185527; doi:10.1186/1471-2148-5-39)
Supplement: Additional File 2 — A rich text format file containing the Clustal W amino acid sequence multialignment of PSG N1, N2 and N3 domains. The RGD-like motif is boxed for comparison between domains. [file 1471-2148-5-39-S2.rtf]

                10        20        30        40        50        60        70
                 |         |         |         |         |         |         |
17N1    SLLSCCLLPTTARVTVEFLPPQVVEGENVLLRVDNLPENLLGFVWYKGVASM-KLGIALYSL-QYNVSVT
25N1    SILTYWLLPTTARVIIHSLPLQVVEGENVLLHVYNLPENLLGLAWYRGLLNL-KLGIALYSL-QYNVSVT
19N1    SLLTCWFLPITARVTIESVPPKLVEGENVLLRVDNLPENLRVFAWYRGVIKF-KLGIALYSL-DYNTSVT
22N1    SLLTCWLLPITAGVTIESVPPKLVEGENVLLRVDNLPENLRVFVWYRGVTDM-SLGIALYSL-DYSTSVT
26N1    SLLTCWFLPTTARVTIESLPPQVVEGENVLLRVDNMPENLLVFGWYRGMTNL-RQAIALHSL-YYSVTVK
28N1    FLLTCWHLPTTARVTIESFPPQVVEGENVLLRVDNMPENLLVFGWYRGMTNL-RHAIAL----YYSLTAK
18N1    SLLTCWLLPTTARVTIESLPPQVYEGENVLLRVDNMPENLLVFGWYRGMTNL-WQAIAQHWLYYYSVMVK
24N1    SLLTCWLLPTTTQVDIESLPPQVVEGENVLLRVDNLPENLLGFIWYKGVTDM-SLGIALYSL-TYSRGVT
20N1    SLFTCWLLSTTAKVTIHS-PLQVVEGQNVFLRVDNLPEDLLAFAWYRGLRNW-RVAIALHLV-EYNASMT
27N1    SLFTCWLLSTTARVTIHS-PLQVVEGENVLLRVDNLPENLLAFSWYRGLKNW-QLAIALHLL-DYNTSMT
21N1    SLLTCWLLSTTASVTIQS-PKHVVEGENILLQVDNLPENLLAFAWYRGLINW-RLTIALHFL-DYSTSMT
23N1    SLLTCWLLSTTASVTIQS-PQHVVEGENILLQVDNLPENLLAFAWYRGLTNW-RLTIAVYLL-DYSTSMT
29N1    SFLTCWYLSTTSKVTIELLPSQVVEGEDVLFLVNNLPGNLTAFAWFKGRTNR-KHGIALYA-VASDLYV-
32N1    FLLTSWFLPTTVQVTIELVPPQVAEGENVLIIVYSLPEDLTAIAWFKGVTNM-NLGIALYA-LASNISVK
30N1    SFLTCCHLPTTAQITIELEPPQVIEGENVLIRVNNLTENLITLAWFRGMRIK-SPQIGQYT-PATKVTVL
31N1a   SFLTCCHLPATAQITIELVPPHVIEGENVLIRVNNLPENLTTLVWFRGMRIK-SPQIGQYT-LATNVTVL
16N1    SLLACWLLSTTAQVTIESVPFNVVEGENVLLRVDNLPENLITLAWYRGLR-----KIVVYTL-NTKVSVM
29N2    FLWNCGRLVTSSQPRIESFPSIVTEGQHVILHVYNIPENLQGFIWFKGMTVHRHLEIGRYTI-GRKSSVF
26N2    SLFICGRPTTLVGPTIELVPASVAAGGSVLLLVHNIPKYLQSLFWYKGLIVFNKVEIARYRR-AKKSRES
28N2    SLFICGRPTTLEGPTIELVPTSVAAGGSILLLVHNIPKYLQSLFWYKGLIVFNKVEIARYRR-AKKSREP
18N2    SLFICVRPTTLISPTIELVPASVAAGGSILLLVHNIPKYLQSLFWYKGLIAFNKVEIARYRT-AKNSGEP
17N2    SLFICERPTTLVPPTIELVPASVAEGGSVLFLVHNLPEYLISLTWYKGAVVFNKLEIARYRT-AKNSSVL
19N2    SLFICGRPSPPALLTIESVPASVAEGGSVLLRVHNLPEHLQSLFWYKGLTMFNKVEIARHRT-AKNSIEM
22N2    SLFICGRPSPPALLTIESVPASVAEGGSVLLLVHSLPDNLQSLLWYKGLTVFNKVEIARHRT-VKNSSEM
25N2    STFICGHPFFPAKLTIESVPPSVAAGGSVLLRVHNLPEHLQSLFWYKGLIVFNKVEIARYRT-AKNSSEP
21N2    SHFTCGRPSFPAKLTIESVPPRVAEGGRVLLRVHNLPEYLQLFFWYKGVIMIHKVEIVRHRT-LKNLSDP
23N2    SHFTCGRPSFPAKLTIESVPPSVAEGGSVLLRVHNLPEYLQLFFWYKGVIMIHKVEIVRYRT-LKNLSDP
27N2    SHFTCGRPSFPAKLTIESVPPRVAEGGSVFLRVHNLPEYLQLFFWYKGVIMTNKVEIVRHRT-LKNLSDP
20N2    ALFTCGRPTSPAKLTIESVPPRVAEGGSVLLLVHNLPEYSQLFSWYKGLTLFNEVEIAEYKI-AKNLSDP
16N2    SLFICGRPSFPAKLTIESVPPSVAEGGSVLLRVHNLQDKLRGLSWYKGAHVSRNLEIARQII-AKNSSVP
24N2    SILTCGRPPTSAQLSIESVPPSVAKGESVLLLARNLPENLRAIFWYKGAIVFKNLEVARYVI-AKNSSVL
32N2    FFWTCGPLSPSAQLTIESVPPKVAEGGSVLLVVHNLQGNLRSLFWYKGMIVSRNLEVARHII-DTNLSVH
30N2    SLWTCEHPSPHAKLTIESVPPGISEGGSVLLLVKNLPQNLLSLFWYKGVIAVKKFEIARHIK-ATNSSVP
31N2    SLWTCEHPSPHAKLTIESVPPGISEGGSVLLLVKNLPKNLLSLFWYKGVIAVKKFEVARHIK-ATNSSVP
16N3    SLSSCCDTLDSTQLIIDPMPRYAAEGESILLRVLNLPEDFQVFCWYKGALIFQIFKIAEYSR-ARNSITK
24N4    SFSTCCDPLASAPLTIDPVPQQAAKGENVLLQVHNLPEDLRMFSWFKSMYSTQIFKIAEYSR-AFNSVIR
18N3    SHSLCCDTLDSAQLSIDPVPRHAAEGGSVLLQVHNLPEDVQTFSWYKGVLSTQDFKIAEYSI-ATKSIIR
26N3    SLSLCCDTLDSAQLSIDPVPQHAAEGGSVLLQVHNLPEGLQAFSWYKGVLSTQDFKIAEYSI-ATKSIIR
28N3    SLSLCCDTLDSAQLSIDPVPQHAAEGGSVLLQVYNLPEGLQTFSWYKGVLSTQDFKIAEYSI-ATKSIIR
25N3    PLSLCCDTLDFAQLSIDPVPRYAVEGGSVLLQVHNLPEDLQTFSWYKGVHNTHGFKIAEYSI-ATKSIIS
17N3    SLSSCCHPLDSPQLSIDPLPPHAAEGGRVLLQVHNLPEDVQTFSWYKGVYSTILFQIAKYSI-ATKSIIM
21N3    ---PCCDPLDSAQLRIDPVTPHAAEGESVLLQVHNLPEDLQTFSWYKGVDSTPSFQIVEYSK-AMKSIIS
23N3    ---PCCDPLDSAQLRIDPVTPHAAEGESVLLQVHNLPEDLQTFSWYKGVDSTPSFQIVEYSK-AMKSIIS
20N3    ---PCCDPPDSARLRIDPVTCHAAEGGSVLLQVHNLPEDVQTFSWYKGVDSTPYFRIVEYSK-AMKSIFS
27N3    SLSPCCDTLDSAQLRIDPVTRHATEGESVLFQVYNLPKDPQAFSWYKGVDSNPYFKTVEYSK-ARYSMLI
19N3    SLSSCCDAFNSVQLRIDPVPPHAVEGESVLLQVHNLPEDVQTFLWYKGVYSTQDFKIAEYSI-VTESIIS
22N3    SLSSCCDDFNSVQLRINPVPPHAAEGERVLLQVHNLPEDVQTFLWYKGVYSTQSFKITEYSI-VTESLIN
32N3    SLSPCCNPLTSSQLMIEPVPRYAVEGESVLFMVHNLPKDLQTFSWYKSVYGAEILKITEYSR-AMSSTTR
30N3    YLLTCYHPL---QVKIESLPQNVAVGKTVLLLVHNLPEDFQAFFWYKSAYRRDTYKIAEYKR-AMDATIL
31N3    SLSTCYQLS---QVKIESLPQKVAVGKSVLLLVHNLPEDFQAFFWYKSAYRRDTYKIVEYSR-AMDTTIM
29N3    PAFQCCNPSTSSKLMVEAVPRYVAEGESVLLLVHNLPEELISFTWYNSMYRVPAFKIVEFNV-IRNITTW
                       :.  .     *  ::: . .:      : *:..                      

 

               80        90       100       110       120
                 |         |         |         |         |
17N1    GLKHSGRETLHRNGSLWIQNVTSEDTGYYTLRTVSQRGELVSDTSIFLQVY
25N1    GPEHSGRETLHRNGSLWIQNVTQEDTGYYTLRTISKNGKLESNTSMFLQVY
19N1    GPEHSGRETLHSNGSLWIQSATREDTGYYTFQTISKNGKVVSNTSMFLQVY
22N1    GPKHSGRETLYRNGSLWIQNVTREDTGYYTLQTISKNGKVVSNTSIFLQVN
26N1    GLKHSGRETLYINGTLWIQNVTQEDTGYYTFQTISKQGEMVSNTSLYLHVY
28N1    GLKHSGRETLYINGSLWIQNVTQEDTGYYTFQTISKQGEMVSNTSLYLHVY
18N1    GLNHSGREILYINGSLWIQNVTQEDTGYYTFQTISKRGEIVSNTSLYLHVY
24N1    GPVHSGRETLYRNGSLWIQNVTQEDTGFYTLRTISKRGEIISNTSMHLHVY
20N1    GPEHSDREILHSNGSLWIQNVTQEDTGYYTLQTISKHGKLVSNTSTFLQVY
27N1    GPDHSDREILYSNGSLWIQNVTKEDTGYYTLRTISKHGELVSNTSTFLQVY
21N1    GPEHSDREILYSNGSLWIQNVTQEDTGYYIFQTISNHGELESNTSTFLQVY
23N1    GPEHSDREILYSNGSLWIQNVTQEDTGYYTLQTISNHGELESNTSTFLQVY
29N1    ---HSDRETLYNNGSLMIHNVTQKDRGYYTLRTFNKHAETVSTTFTFLHVN
32N1    GPEHSGRETVFSNGSLLLHNVTQKDTGFYTIRTLNRHGKIVSTTSIYLHVY
30N1    GPGHSGRETLYSNGSLQIYNVTQEDIGFYSLRIINKHAEIVSITSIYLNVY
31N1a   GPGHSGRETLYSNGSLQIYNVTQEDIGFYSLRVMNRHGKIVSITSIYLNVY
16N1    GQMYSGREIVSSNGSLWIHNVTRKDTGLYTLRTVNRRGEIVSTS-------
29N2    GPAYSGREKLDSNGSLRIENVTQKDAGLYTLRVLGTDMKSEE-AHVELQVN
26N2    GPAHSGRETVYSNGSLLLQNVTWKDTGFYTLRTLTRYQKMEF-AHIYLQVD
28N2    GPAHSGRETVYRNGSLLLKNVTWKDTGFYTLRTLTRYQKMEL-AHIYLQVD
18N2    GPAHSGRETVYSNGSLLLQNVTWKDTGFYTLRTLTRYQKMEF-AHIYLQVD
17N2    GPAHSGRETVFSNGSLLLQNVTWKDTGFYTLRTLNRYPRIEL-AHIYLQVD
19N2    GPAHSGREIVYSNGSLLLQNVTWKDIGFYTLRTLNRYSRIEL-AHIYLQVD
22N2    GPAYSGREIVYSNGSLLLQNVTWEDTGFYTLQIVNRYWKMEL-AHIYLQVD
25N2    GHAHSGRETVYSNGSLLLQDVTWKDTGFYTLRTLNRYRKMKL-AHIYLQVD
21N2    GPAHSGREIVFSNGSLLLQNVTWKDTGFYTLQTVNGFREMEL-AHIYLQVE
23N2    GPAHSGREIVYSNGSLLLQNVTWKDTGFYTLQTVNRYWKMEL-AHIYLQVD
27N2    GPAHSGRETVFSNGSLLLQNVTWKDTGFYTLQTLNRYRKMEL-AHIYLQVD
20N2    GPAHSGREIVFSNGSLLLQNVTWKDTGFYTLQTVNGFREMEL-AHIYLRVD
16N2    GPAHSGRETVYSNGSLLLQNVTRNDTGFYTLQTLSRHRKMEL-AHVQLQVD
24N2    GPAHSGREIMYSNGSLVLQNVTRNDAGFYTLRTLSTDLKAEV-AHVQLQVD
32N2    GPLHSGRETIYSNGSLMFYNVTWKDSGLYTLRTLSTDMKTEL-AHVQLQVD
30N2    GPAHTGRETVFSNGSLLLQEVMQSDTGFYTLRTMSTDLKDEV-AHVQLYMD
31N2    GPAHTGRETVFSNGSLLLQEVMQSDTGFYTLRTMSTDLKDEV-AHVQLYMD
16N3    GPAQSRTERVYTNGSLLLQDVTEKDTGLYTLQTIDRNFKIEK-AHVQIQVN
24N4    GPAHSRREIVYTNGSLLLQDATEKDTGMYTLQTVDRNFKIET-AHVKIQIN
18N3    GRAHSRREIGYTNGSLLLQDVTEKDSGLYTLITIDSNVRVVT-AHVQVNIH
26N3    GRAHSRREIGYTNGSLLLQDVTEKDSGLYTLITIDSNVRILT-AHVQVNIH
28N3    GRAHSRREIGYTNGSLLLRNVTEKDSGLYTLVTIDSNMRVVT-AHVQVNIH
25N3    GRAHSRREIGYTDGSLLLQDVTEKDSGLYTLIAIDSNVRVVR-AHVQVNVH
17N3    GYARSRRETVYTNGSLLLQDVTEKDSGVYTLITTDSNMGVET-AHVQVNVH
21N3    GSAYSRREIGYTNGSLLLQDVTEKDSGLYTLVTIDSNMRVET-VHVQVNIY
23N3    GSAYSRREIGYTNGSLLLQDVTEKDSGLYTLVTIDSNMRVET-VHVQVNIY
20N3    GYAHSRRETGYTNGSLLLQDVTEKDTGFYTLLTIDSHVKVET-VHAQVNVH
27N3    GQSYSRREIGYLNGSLLLQDLTEKDSGLYTLITIDSNVKVET-LHVQINVH
19N3    GRAHSGREIGYTNGSLLLQDVTEKDSGFYTLVTIDSNAKVET-AHVQVNVN
22N3    GYAHSGREILFINGSLLLQDVTEKDSGFYTLVTIDSNVKVET-AHVQVNVN
32N3    GSELKRRERVYTNGFLLLQNATEKDAGMYILETLSRDFKIEK-AQVQLYVN
30N3    GSAYSSREFIYNNGSMLIIDVTEDDAGYFLLEILREDLKIEK-AYIQLHVN
31N3    GSAYSLREFIYNNGSMLIIDVTVDDAGFFMLEILRKDFKIEK-AYIQLHVN
29N3    GDVYRGRDTVYANGSLMLQDVTEEDARMYTLETLNVNYTVER-AHVQFYVN
               :    :* : : .   .*   : :                     
